# Exploring the Framing of Animal Farming and Meat Consumption: On the Diversity of Topics Used and Qualitative Patterns in Selected Demographic Contexts

**DOI:** 10.3390/ani8020017

**Published:** 2018-01-24

**Authors:** Hanneke J. Nijland, Noelle Aarts, Cees M. J. van Woerkum

**Affiliations:** 1 Strategic Communication Group, Wageningen University, Hollandseweg, 1, 6706 KN Wageningen, The Netherlands; Noelle.Aarts@ru.nl (N.A.); ceesvwoerkum@gmail.com (C.M.J.v.W.); 2 Hanneke J. Nijland Research & Consultancy, Pootakkerweg 10, 6706BX Wageningen, The Netherlands; 3 Institute for Science in Society (ISiS), Radboud University, Heyendaalseweg, 135, 6525 AJ Nijmegen, The Netherlands

**Keywords:** animal farming, meat consumption, framing, topics, taste, human health, animal welfare, environmental impact, contextual influence, complexity

## Abstract

**Simple Summary:**

In various contexts, people talk about the farming and consumption of animals using different arguments to construct and justify their (non-)acceptability. This paper reports on a qualitative research among consumers with different backgrounds in urban and rural areas in The Netherlands and Turkey. We present an elaborate methodology for qualitatively researching everyday-life talk about animal farming and meat consumption. We explain how we collected and organised topics people refer to, and looked at the possible relation of complete argumentations with the researched contexts. The resulting long list of topics includes animal welfare arguments, but shows that in everyday-life many others are used, such as health, taste, money, religion, and environmental impact. Our research indicates several ties between mentioned topics and the researched contexts—the most noticeable pattern being the difference between respondents in cities and rural areas. However, in contrast to what literature suggests, single contextual features, like country or gender, offered relatively little insight into the differences that showed up in the complete argumentations. This, we argue, does not imply that context does not matter, but rather that so many cultural and personal contextual aspects play a role that singular contextual features cannot sufficiently explain framing.

**Abstract:**

In various contexts, people talk about animal farming and meat consumption using different arguments to construct and justify their (non-)acceptability. This article presents the results of an in-depth qualitative inquiry into the content of and contextual patterns in the everyday-life framing regarding this issue, performed among consumers in various settings in two extremes in the European sphere: the Netherlands and Turkey. We describe the methodological steps of collecting, coding, and organizing the variety of encountered framing topics, as well as our search for symbolic convergence in groups of consumers from different selected demographic contexts (country, urban-rural areas, gender, age, and education level). The framing of animal farming and meat consumption in everyday-life is not a simple one-issue rational display of facts; people referred to a vast range of topics in the categories knowledge, convictions, pronounced behaviour, values, norms, interests, and feelings. Looking at framing in relation to the researched demographic contexts, most patterns were found on the level of topics; symbolic convergence in lines of reasoning and composite framing was less prominent in groups based on single demographic contexts than anticipated. An explanation for this lies in the complexity of frame construction, happening in relation with multiple interdependent contextual features.

## 1. Introduction

The farming and slaughter of animals and the related consumption of meat are increasingly being contested in our current society [1,2,3,4]. However, the ways in which (non-)acceptability of the farming and consumption of animals is constructed are not univalent. The world consists of a variety of (sub-)cultures, in which different arguments are applied by people with different values, norms, and interests, to different animals [1].

Previous research into various aspects of the human–animal relation shows, that sensitivity to context is of great importance [5,6]. Research in Western countries—mostly focusing on animal welfare perception—indicates that contexts within societies, such as urbanization level, cultural value systems, the kind of production system under scrutiny, group membership like gender or age, and personality traits such as empathy, influence the perception of animal farming and meat consumption [1,3,7,8,9,10,11,12,13,14,15,16,17,18,19,20]. The relationship to and species of animal involved also effects the construction of acceptability: dogs, for example, are commonly only kept as pets in Europe and are a taboo to eat, while chickens and rabbits are a commonly accepted species for consumption (though the latter is also kept as a pet) [21,22]. In addition, there is evidence of varying perceptions of animal husbandry between countries [15]. In Western European countries for example, societal concerns regarding the use of farm animals are a hot topic. Environmental impact of animal farming and meat consumption is increasingly recognised, the European Union has a separate General Directorate for animal welfare and several scientific studies have been performed in member states on issues related to the perception of animal welfare [1,3,7,8,9,10,11,12,13,15,16,17,23,24,25,26,27,28]. In the Netherlands, a country with a rich background in animal farming (mostly intensive large-scale, though depending on the type of animal that is being farmed, professional farming systems in the Netherlands can be divided into conventional, enriched/free-range, organic, and organic-dynamic), a political party was established to represent the voice of animals even occupies two seats in parliament, making it one of the leading countries in Europe in terms of problematising animal husbandry. Eurocentric ethical concerns and research results however cannot easily be extended to EU membership candidates like Turkey, since geography, production systems, economic/technological development, but also socio-cultural and moral standards vary [2,15,29]. Animal husbandry in Turkey consists of traditional small-scale systems, next to an increasing number of large-scale confinement systems (the share of organic animal agriculture in Turkey, though it is on the rise, is still relatively small) [30,31]. Though Turkey currently strives for a more innovative and competitive rural economy that complies with the EU’s Acquis communautaire, Turkish legislation controlling the handling of farm animals hardly exists, and scientists addressing reform and sustainability of animal agriculture in Turkey have not yet focused on any aspect of socio-cultural acceptance [31,32,33,34,35]. However, part of a pilot research on animal welfare perception that the main author performed in Turkey in 2004 showed broadly ranging views: from being mesmerized with modern farming and slaughter systems and putting big importance on meat eating, to feeling disgust about animal production and consumption [36].

Two main gaps can thus be distinguished in the understanding regarding the framing of animal farming and meat consumption. Firstly, most studies regarding the perception of the production and consumption of animals have concentrated on single issues, such as animal welfare and willingness to pay [1,3,7,8,9,10,11,12,13,15,16,17,18,19,20,23,24,25,26,27,28]; while, when made explicit, social acceptability of animal farming and meat consumption may encompass many more aspects, ranging from, for example, nutritional value, consumer safety and environmental impact, to meat taste, religious views, aesthetics, and cultural and personal habits [3,11,28,37,38,39]. Secondly, though it is clear that there is a connection between context and aspects of the human–animal–environment relation, research has mainly been performed in Western contexts. At the onset of this research, there was no research on the subject in Turkey (besides the pilot executed in 2004), a country that as an important trade partner and candidate to the European Union does belong to the European sphere, but contextually differs from Western European countries such as the Netherlands in many aspects: geography, culture, religion, farming styles, perception of the importance of animal welfare [40,41].

This suggests that there are multiple ways to construct various aspects of animal farming and meat consumption in terms of (non-)acceptability, and that these are likely related to contextual factors of geographical, cultural, and personal nature. The apparent pluralism in determining which arguments should be or are decisive, may give space to the legitimation of different behaviours [42]. To enable effective communication, for example between science, industry, and consumers, or to design effective and context-transcending policies for sustainable agriculture, it therefore is essential to distinguish and contextualise similarities and differences in consumer framing regarding the (non-)acceptability of animal farming and meat consumption [15,43]. To develop a better understanding of this framing and encourage societal dialogue based on scientific comprehension, we designed a case-driven in-depth interpretive research carried out amongst consumers from various backgrounds in urban and rural areas in the Netherlands and Turkey. The research was centred around the question: *What frames are used in everyday-life to construct (non-)acceptability of farming, slaughter and consumption of animals, and how do these relate to context?*

In this article, we describe the analytical steps of coding and organizing the variety of encountered topics, and distinguishing qualitative patterns in the framing of animal farming and meat consumption in selected demographic contexts—and present the main results.

## 2. Materials and Methods

### 2.1. Conceptual Framework

We live in a world that is differently understood. Related to this, people can talk about events or phenomena in numerous ways. Whether consciously or not, in interaction people choose specific descriptions of reality out of innumerable possible descriptions, in order to accomplish various goals [44,45,46,47]. A key concept to approach the specific ways people address an issue, that is acknowledging of this selective and context-dependent nature of communication, is the *framing* metaphor.

In literature, divergent definitions of framing can be found, whether explicit or inferred by usage. Besides viewing framing as semiotic behaviour (“message framing” or “frame construction”), the term has been used to define the cognitive schemas of interpretation, mental filters or “mindsets” through which we perceive reality and that guide our action, the process of fitting new information into one’s mindsets (“sense-making”), or combinations of these [48,49,50,51,52,53,54]. We have chosen to use framing to refer to the communicative behaviour that is the result of these inner filters and processes *as well as* the interaction with others. This view includes but also transcends the idea that the narratives brought forward in conversations (including those about animal farming and meat consumption) are representations of some more or less stable cognitive building block-like structure: though what is said relies on what is cognitively available, frames are dynamic and flexible presentations aimed at pursuing specific goals in a specific context, making use of cognitions, that iteratively get added to and continuously change while we communicate with (real or imaginary) others and our physical surroundings [55,56]. Framing in this research thus is defined as the dynamic way people selectively and strategically use (or hide) available cognitions to narratively present a situation or action in interaction—or as Aarts and Van Woerkum [44] captured it in more detail: “frames are constructed and legitimated in interaction by combining and integrating cognitive building blocks referring to previous experiences, expectations and objectives concerning the issue at stake (content), the actors involved (relations) and the process that takes place (process)”. This chosen delineation of framing reflects an integration of so called cognitive approaches (emphasizing that frames are representations of cognitions stored in memory) and interactional approaches (focusing on the dynamic enactment of frames in on-going interaction) [50].

Of course, the complete frames that are brought forward in conversations and documents amount to more than the sum of their parts: they are formed by combining arguments that mutually influence one another, are interrelated, and dynamic in direction and meaning. Still, the first analytical step on the way to understanding these complex frames encompasses breaking them down into the content elements they are made up of, and then look for patterns. An important existing tool to distinguish the content of and patterns in framing is the model of the *frame-of-reference* [1,6,57,58,59]. The term refers to the cognitive filter through which we interpret situations, or in other words: the cognitions within consciousness that we use and “refer to” when we frame something. The main idea of this concept is that people’s perceptions of a certain issue are the result of a (largely unconscious and rather fluid) process of tuning of the cognitions that are part of their frame-of-reference, which is reflected in the communicative behaviour displayed [6]. The name “frame” may be confusing with regard to the way we conceptualised framing earlier on. However, the model of the frame-of-reference is of great importance to the researching of patterns in framing, because it offers clear and workable categories that help probing for and organising topics. A common way [1,6,57,58] to organise the elements of the frame-of-reference regarding a certain issue, is to distinguish between:

- *Values*: opinions about what is intrinsically important;
- *Norms*: translation of values into rules of conduct;
- *Interests*: including material (economic) as well as immaterial (social, moral) interests;
- *Knowledge*: constructed out of experiences, facts, stories, and impressions;
- *Convictions*: opinions about “the way things are”, assumptions that are taken for granted.

According to the original model of the frame-of-reference, the interplay between topics within these five categories determine people’s perceptions and framing of a certain issue. However, when attempting to code pilot interviews for the current research, we found we needed to fine-tune this list of categories. First of all, the original grouping of the categories of cognitions belonging to the frame-of-reference in our view is limited, because it seems to reduce cognitions to thoughts only. Though often omitted from cognitive models, feelings are known to play a determining role in framing [60,61,62,63,64,65]. Because feelings are intertwined with linguistic thought (feelings can emerge after entertaining a particular thought, and similarly, we sometimes conceptualise or rationalise earlier felt emotions, states, or physical sensations), they could be argued to already be in the model, being two sides of the same coin. However, people in conversations—and especially regarding a topic like animal farming—specifically *refer* to feelings, in addition to referring to their thoughts regarding values, norms, interests, knowledge, and convictions. We thus opted to add “feelings” as a separate category to the model. In a similar line of thought, because people consistently refer to behaviours in conversations while this was not yet accounted for in the model, we added the category “behaviours”. Finally, we chose to collapse the originally separate categories “knowledge” and “convictions” into one category, due to the undefinable grey area between fact and opinion [65,66]. Because in framing they both concern expressions of thoughts about “the way things are” (or what Ford [56,67] calls “second order reality”), knowledge and convictions share a much higher degree of resemblance and overlap than the other categories. Especially in topics regarding farming, slaughter, and consumption of animals, it is fairly difficult to differentiate between conviction and “true” knowledge (even much scientific knowledge is conflicting, as outcomes depend on context and perspectives) [68,69]. Within the scope of the current research, it therefore made more sense to combine them into one category. Besides these category adaptations, to contribute to more clarity and less chance of overlap, we refined the names and definitions of the existing categories. In the current research, when searching for patterns in framing, we accordingly distinguished between:

- *Behaviours*: what is done: pronounced personal past and present actions, including habits and exceptions;
- *Values*: rational concerns: conceptualisations about what and whom is considered important and to what extent;
- *Norms*: what is brought forward that should be done: ideal rules of conduct imposed on the self—and possibly others;
- *Feelings*: affective concerns: physical sensations, states, and emotions (while framing often accompanied by gestures and facial expressions);
- *Interests*: recognised stakes and goals that inner drives motivate us to strive for, both material (physical, economic) as well as immaterial (social, moral, aesthetical);
- *Knowledge and convictions*: opinions about the way things are, about (self-)efficacy and the effects certain situations will have, associations and assumptions about what is true, including the perceived behaviours, values, norms, feelings, interests, and knowledge and convictions of others.

While all categorisation is artificial and ambiguous, and the discerned elements obviously relate to and mutually influence one another, these categories offers a means to organise framing content in a meaningful way. The frame-of-reference is a proven tool to probe for the various cognitions and topics that constitute the content of lines of reasoning and composite frames regarding a certain issue, as well as to organise this content. Additionally, when data from conversations with multiple persons are combined, an overview of framing elements related to a specific issue (here: animal farming and meat consumption) can be composed, can be used as a checklist and provide insight into topics (or whole categories) that are left *out* of the framing (cf. Hoffmann’s “Boundary setting” [51]). As will be explained in Section 2.2., in our research, we have probed for cognitions using questions formulated based on the six categories of the frame-of-reference, coded the frames that were brought forward down to the level of topics, and organised these under the six main categories, adding using sub-categories for clearer organisation.

To get more insight into the contextuality of and interrelation between frames, the *symbolic convergence theory* [70] is of use. Symbolic convergence refers to similarities that occur in communicative behaviour, for example in certain groups of people or contexts. People use language to construct stories to give meaning to the world around them, and by sharing interpretations in groups of people, a structure is created and language and stories may converge into a shared story. Thus, a set of socially shared and commonly used narratives for a group—in other words: “common frames”—can be differentiated [6,70,71]. This is not to say that these stories are rigid, or that they are all exactly the same or connected by one essential common feature—common frames can be distinguished based on family resemblances [72]: i.e., connected by a series of overlapping similarities, where one feature may not be common to all of the groups. Accordingly, symbolic convergence can be based on similarities in, for example, the use of topics (listed as codes within the (sub-)categories of the frame-of-reference), certain (his)stories, or composite lines of reasoning leading to constructing a degree of acceptability of the farming, slaughter, and/or consumption of animals. In this research, we looked for common frames to find patterns in the different sets of contextual factors that were distinguished (see Section 2.2.: Research Design).

### 2.2. Research Design

The methodological approach of our research is a qualitative framing analysis—driven by the case of animal farming and meat consumption. Finding and contextualizing content and patterns in everyday-life consumer framing of the production and consumption of animals starts with collecting and breaking down frames that are used by different persons in different settings. Based on this, we can get an idea of the topics that are or can be involved, and whether common frames occur in relation to demographic contextual features. Our data consists of 50 semi-structured in-depth conversations with consumers in various settings, added upon by encountered documents and unplanned informal conversations (the data used to perform this research—documents and (anonymised) conversation transcripts in Turkish and Dutch—can be made available upon request).

Literature indicated that contextual features, such as geography, gender, age group, and education level, are linked to perception and behaviour regarding several aspects of animal farming and meat consumption (in many cases: animal welfare) [1,3,7,8,10,11,12,13,14,15,17,18,19,24,73]. To select respondents for the 50 semi-structured in-depth conversations that form the key source of data in our research, we followed a target-group oriented theoretical sampling regime [73]. For the conversations (both planned and unplanned), within each of the two countries, an urban and a rural region was appointed, forming four case-study areas: the city centres of the Dutch and Turkish capitals Amsterdam and Istanbul; and the rural area located in the Dutch provinces of Drenthe/Overijssel and in the Turkish provinces of Aydın/Balıkesir. These locations provided ample area for research, while still being comparable in terms of the availability of both animal and non-animal protein sources (in contrast to, for example, Turkish provinces in Middle and Eastern Anatolia, where there is not always a choice). Respondents to the planned conversations were further selected on the basis of gender (male; female), age group (15–30; 30–50; 50–70), education level (high; middle; lower; student), and differing pronounced protein consumption behaviour (meat eater; vegetarian; vegan; compromise). The characteristics of the selected respondents for the planned conversations are listed in Appendix A: Table A1.

The selection technique for finding respondents for the planned in-depth conversations was initially fortuitous, and moved on to chain referral (popularly known as the “snowball” method) [74]. This meant that in the onset, we approached random people in the designated case-study areas and asked for their cooperation, and when this did not render sufficient people fitting a specific group of criteria, we moved on to asking local people to refer us to someone fitting those criteria who would be interested to engage in a conversation with the first author. Selection of respondents for the planned conversations continued until theoretical saturation [75]—that is: until no new information/frames were found in the last four to five conversations. The originally planned number of conversations was 30 (15 in each country), but, because new data was still emerging, we continued to select people. Ultimately a total of 50 in-depth interviews (25 in each country) were performed.

The data selection technique we applied is called target-group oriented theoretical sampling [73]: we selected data from sources that expectedly provide good coverage over the main case-study areas, the Netherlands and Turkey, yet were picked to differ as much as possible from one another on a range of measures. With this technique we did not aim to representatively capture all possible variations or average reactions of groups of people within the European context, but rather used an accumulation of unique cases [76] to gain a deeper understanding of each of those unique cases, as well as of the more general aspects of the framing of animal farming and meat consumption. The applied sampling method thus does not allow for making statistical inferences about groups of people or context-dependency; however, it is well suited for distinguishing patterns in the construction of frames, and providing plausible explanations for them [77].

**Figure 1:**
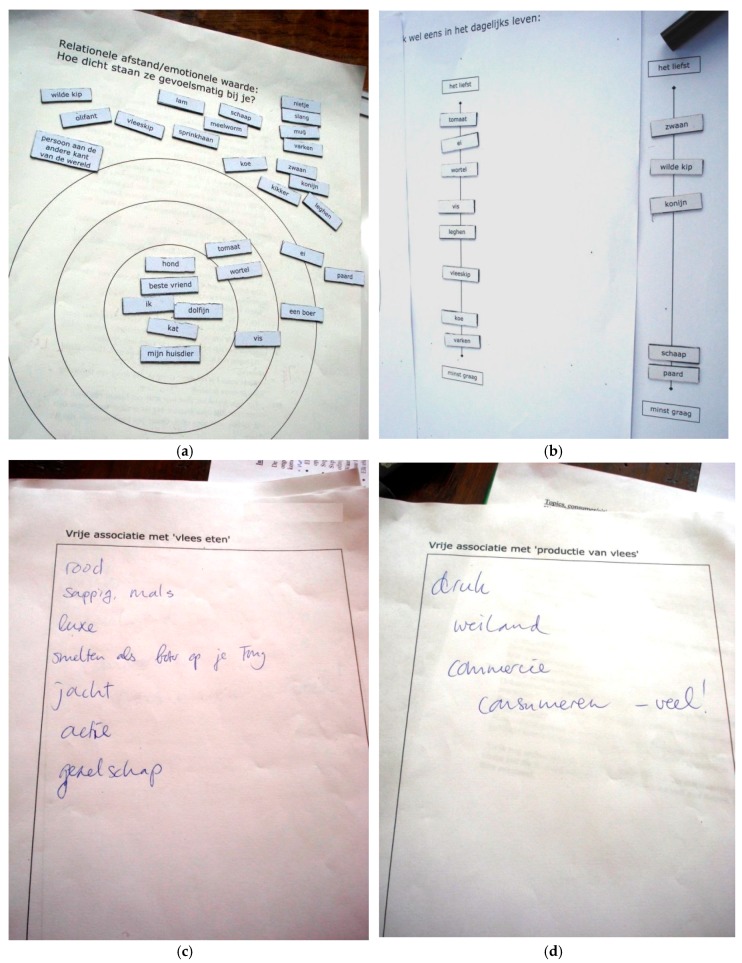
Example results to illustrate our visual interview techniques: (**a**) Circles of relational distance/emotional value: “How close do they feel to you?”, using the items presented in Table 1; (**b**) Lines of items eaten in daily life vs in exceptional cases; (**c**) Free-listing on the topic “eating meat” (“*Red; Juicy; Tender; Luxury; Melting on your tongue; Hunt; Action; Company*”); (**d**) Free-listing on the topic “production of meat” (“*Busy; Meadow; Commerce; Consumption—a lot!*”).

The planned conversations, all of which were performed on location by the main author in the native language of the respondents in 2010, followed the same semi-structure, and lasted about 1–2 hours each. Confidentiality was offered and permission to record was asked. Since the subject under scrutiny is known to be rather delicate (anger-, discomfort- or defensiveness-evoking) for some people, at the onset of the conversation the research was introduced as being about human–animal relations and food culture (rather than the acceptability of animal farming and meat consumption). The structure of the in-depth semi-structured conversations was specifically designed to encourage respondents to grow curious and self-analytical, rather than defensive. The conversations started with a visualisation technique, asking respondents to intuitively place magnetic patches containing descriptions of humans and different animals in relation to inanimate objects on the circles of Wenz [78], determining relational distances (see Table 1 for the list of items and Figure 1a for an example result). This rapid appraisal method [79] functioned as an ice-breaker, as well as providing valuable insights that formed a trigger for further conversation. The items were selected to include several non-human animals that are conventionally seen as consumption animals, pets and pests, some human beings, as well as some edible and inedible objects. We moved on to ask whether they could move the items from the circles they ate on a daily basis and in exceptional cases, on two lines ranging from “gladly” to “rather not” (Figure 1b). Letting our conversation partners analyse themselves, with regard to the reasons they have for the distinctions they make, proved effective in getting to the issue, without imposing topics or ambivalent feelings. Subsequently, free association listing on the topic “eating meat” (Figure 1c) and, later on, on the topic “production of meat” (Figure 1d), lead to many themes and arguments related to the framing of animal farming and meat consumption.

We followed an interview method called laddering, which entailed asking why-questions, allowing people to elaborate on their previous answers until no deeper clarifying answer could be given [80,81]. Furthermore, we used the model of the frame-of-reference to formulate questions that covered the complete range of topics that may play a role in the framing of animal farming and meat consumption, without putting the exact topics or arguments in people’s mouths. To further ensure that our interpretations indeed are credible approximations of frames, we summarized our interpretation of our conversation partners’ arguments, and asked for feedback to see if the interpretation resonated with their personal views regarding the topic.

The planned in-depth conversations were triangulated [73] with encountered documents and unplanned informal conversations. These documents consist of several hundreds of newspaper articles, social media posts, fora conversations, and artwork related to the subject of breeding, slaughtering, and consumption of farm animals, that were collected in the Netherlands and Turkey (in Dutch, Turkish and English) between 2004 and 2014. The unplanned informal conversations refer to dozens of conversations on the topic that were overheard, partaken in or initiated, in the Netherlands and Turkey between 2009 and 2014 (in Dutch, Turkish, and English), of which notes were kept. The data from the collected documents and unplanned conversations were added to the analysis when they contained new or remarkable information. In addition, they were used when they represented typical ways of framing the (non-)acceptability of animal farming and meat consumption.

Though the original data naturally is stored in the original language, the common language for processing, analysis, and reporting was English. We coded the frames that were brought forward in the individual conversations (of which the recordings were transcribed word-for-word) as well as additional documents and notes down to the level of topics, and organised the assigned codes into categories and sub-categories, using the model of the frame-of-reference as a basis for organising this cognitive content of framing, but also generating new sub-categories when these emerged from the data. We coded using Computer Aided Qualitative Data Analysis Software [82]: initially Atlas.ti (version 6.2), but in the course of the project, we transferred to a Microsoft Excel database file for increased overview, with respondents including contextual information in the rows, categories and codes in the columns, and content (i.e., quotes and summaries of frames) in the fields. Using this overview database, we looked whether there were patterns in framing (“common frames”) to be found, in relation to the demographic contexts in which they arose.

## 3. Results

### 3.1. The Diversity of Topics Used to Frame Animal Farming and Meat Consumption

The coded content across the transcripts of individual conversations and documents in our database, accumulated provides a first case pattern through categorization: a systematic overview of the diversity of possible topics that play a role in everyday-life framing of animal farming and meat consumption in the Netherlands and Turkey. The resulting—elaborate but finite—list of topics is provided as List S1: Categories and codes: topics used to talk about animal farming and meat consumption (http://www.mdpi.com/2076-2615/8/2/17/s1). 

#### 3.1.1. General Interpretation of the Use of Topics

Interpreting the listed categories and codes leads to several inferences. First of all, the accrued codes clearly show that the frames consumers used to discuss the production and consumption of animals, touch upon many more issues than just animals and their welfare. Of course, animal oriented topics take up a reasonable portion of the list: “*I just feel bad for the animals. They are used as products, not as living beings.*” (planned conversation, Netherlands, male, 15–30, urban). However, referred-to topics also include human health, nutritional value, meat taste and appearance, social relationships, religion, history, habits, consumption behaviours, global food supply, and environmental impact. By far the largest number of topics that are brought forward concern people and their needs and desires—such as taste and health and getting by financially-, as well as references to the socio-institutional system they have to manage to live in. Examples of these are: “*After I eat meat, I feel stronger.*” (informal conversation, Netherlands, female, 30–50, urban) and “*The intensive farming system developed after the Second World War to prevent hunger from ever happening again. So, it had a good purpose, and now it’s hard to change the way it is.*” (planned conversation, Netherlands, male, 50+, urban). Though the number of topics in a category may not completely coincide with the relative use or importance of said category, the list shows that only a fairly minor share of the topics concern environmental impact.

When performing a framing analysis of ambiguous topics, such as animal farming and meat consumption, the distinction between the framing of empirically observed facts and opinions (that can be constructed out of facts and/or fiction) is not at all that easy to make—which was confirmed during the gathering and analysis of data. For example, the sentence “*My neighbour is a vegetarian*” does not unmistakably show whether this is empirically observed knowledge, hearsay, or an assumption. While there arguably is a difference between “*Science has proven that sows suffer in birth cages*” (reported speech, pointing towards factual information) and “*I believe that sows suffer in birth cages*” (direct speech, using “I believe”, pointing at this being a conviction), the *topics* referred to are the same (in this example: the impact of housing, measures and treatment on animals). Because of this overlap, and the uncertainty in distinguishing factual knowledge from convictions in the analysis of framing, all mentions of thoughts about the way things are were organised into one main category: “knowledge and convictions”. The other key categories that were distinguished were “values”, “norms”, “interests”, “feelings” and “behaviours”.

The length and complexity of the category “feelings” shows that physical sensations, states, and emotions form an integral and complex part of the framing of the (non-)acceptability of animal farming and meat consumption. An example including all three is: “*Though it smells great and I find it really tasty, I don’t eat meat because I feel sorry for the animals. When I think about the way they suffer during farming and slaughter, I get really sad and angry. So even though I love meat, I love the animals more.*” (planned conversation, Turkey, female, 15–30, urban). Not only do people refer to their feelings about the food they consume, or the way animals are reared and slaughtered; they also express how certain values, norms, interests, knowledge and convictions they hold, behavioural decisions they make, as well as feelings they have, make them feel. Examples of this are: “*I feel very strongly about being conscientious, compassionate and consistent in my consumption behaviour. It is why I like who I am.*” (planned conversation, Netherlands, female, 30–50, urban) or the more dissonant: “*I want to change how I feel about killing animals. I think it is natural, but I can’t bear the sight of it. It makes me feel weak. And that doesn’t feel good.*” (informal conversation, Turkey, male, 30–50, urban).

The diversity of codes in the category “behaviours” demonstrates that the commonly made division between “eating meat” and “vegetarianism” is too simplistic when describing consumption behaviour. In the research, most self-named meat eaters still refrained from eating certain species or parts of the animal—“all-meat” consumers are not all that common, and there were also many other “in-between” behaviours, for example, eating less meat or only meat from organic origin. Moreover, a number of people go further than vegetarianism, by not only refraining from meat, but also from other products, such as dairy and eggs. Table 2, originally composed by E. Schurgers [83], added upon by our research findings, illustrates this variety of consumption behaviours.

Pronounced consumption behaviour thus ranges widely: from eating all parts of in principle all animals, via various in-between forms, to vegetarianism and veganism (in more or less strict ways). In the in-depth conversations, we did not only ask to place the animals that were consumed in daily life on a line from “least gladly” to “gladly”, but also, in a similar way, inquired into what respondents would consume in extraordinary cases—for example out of politeness (visiting a foreign culture), curiosity, or necessity (stranded on a deserted island with no food). Personal preferences in taste is an important reason given for variation in behaviour, and all 50 respondents were unique in their preferences and limits to what they would eat. The research technique showed that the species of animal was of great importance to the placement on the lines by individual respondents: “*Chicken, cow, and all is fine, but I would never eat a swan. They are just too noble and beautiful*” (planned conversation, Netherlands, male, 50+, rural), but also that they varied a lot: “*Oh yes, I would have never guessed but swan is actually very tasty. The hunters around here shoot them and apparently, they are just thrown away. But one of our neighbours, a bad ass guy, asked for them and then we had a barbeque!*” (planned conversation, Netherlands, male, 50+, urban). In general, animals that people are unfamiliar with as food and/or that are perceived as pets, are less gladly eaten than animals that are culturally farmed for food. In addition, consumption of foods from certain animal origins appeared to be importantly linked to the situation at hand—what is considered normal, what is available, what is necessary, and when is a situation considered exceptional: “*In the war, we even ate rats. Rats! But, though I’ve heard of people that ate human flesh, I don’t think I could ever do that.*” (planned conversation, Netherlands, male, 50+, urban).

#### 3.1.2. Summary

The content of the frames involves mentions of behaviours, values, norms, feelings, interests, and knowledge and convictions. The diverse (sub-)categories and codes (see List S1: http://www.mdpi.com/2076-2615/8/2/17/s1) that emerged when organising the framing elements within these categories, show that there is a large variety of topics used to talk about animal farming and meat consumption, that can be about oneself, other humans and culture, about the production chain, system and institutions, as well as about (different species of) animals, and environmental issues.

### 3.2. Framing and Demographic Contexts in the Netherlands and Turkey

Looking at the framing of animal farming and meat consumption in relation to the researched geographical contexts (the Netherlands and Turkey; urban-rural areas) and population groups (gender; age group; and education level), we encountered several trends. However, most patterns in framing that we found when looking at these demographic contexts were found on the level of topics; symbolic convergence based on lines of reasoning, and composite framing was less prominent in the separate demographic contexts than anticipated.

#### 3.2.1. The Netherlands and Turkey

Coherent with these topics being on the Dutch political agenda (even the Stemwijzer, the Dutch version of Vote Match, contains multiple questions related to animal welfare and environmental impact.), in the Netherlands animal welfare and environmental impact were often problematized, while in Turkey these were only mentioned by a relatively small share of the respondents. In Turkey, the adverse effects of hormones and especially genetically modified foods (GMOs) were relatively frequently mentioned, linked to human health and naturalness, while our research indicates that in the Netherlands, health is considered more in terms of the effects of red meat on heart disease. Interestingly, the topic of halal meat was more frequently mentioned by Muslim consumers (next to some non-Muslim critics of ritual slaughter) in or visiting the Netherlands than in Turkey; the reason offered was that in Turkey all available meat can be considered halal, while in the Netherlands, you have to specifically look for it. Vegetarianism and veganism was more widespread in the Netherlands than in Turkey (though over the last ten years numbers have increased in both countries) [84,85]. However, these mentioned differences between respondents in Turkey and the Netherlands were on the level of topics—not on the level of composite framing the (non-)acceptability of animal farming and meat consumption.

#### 3.2.2. Age Groups, Education Levels, and Income

The research results indicate that several respondents from higher age groups display distrust in labelling of food products and production practices as part of their framing, such as by telling stories about salesmen fiddling with terms such as organic or free-range: “*I don’t believe what is on the box. Then they say free range chickens, it says so on the box, but you know Hanneke, I don’t believe everything. Because if a chicken is free range it is still inside a cage. Just a bit bigger one. You’re being deceived all the time. Only this week I read a piece in the newspaper that the government does least research of all in the food chain. Because these companies, they have their own logos, but it hasn’t been checked at all. They can just stick it on and that’s not right.*” (planned conversation, Netherlands, male, 50+, urban). Relative to older and lower educated respondents, more younger and higher educated respondents pronounced to opt for meat from alternative sources, flexitarianism or vegetarianism. Another demographic factor (that we did not use to select respondents, but did check for during the conversations) is having an income that allows for the purchase of desired food. Of the respondents, only a homeless person in Amsterdam and a poor farmer in rural Turkey had such limited funds that they could not opt for any alternatives. As the homeless man expressed “*I’ve lived on the street, if you’re hungry you eat everything. If I don’t accept the chicken that is offered to me, I’ll have an empty stomach and it will rot away. That would be absurd!*” (planned conversation, Netherlands, male, 30–50, urban). However, also by many other respondents, economic constraints were a frequently mentioned reason for not buying the ideally desired food. An interesting detail that stood out in this regard was that in the Netherlands alternatives for regular meat were framed as expensive, while in Turkey meat (even regular meat) was brought forward as costly in comparison to meatless meals (meat prices are indeed much higher in Turkey than in the Netherlands [86,87,88]).

#### 3.2.3. Urban and Rural Areas

The most prominent pattern in the use of framing elements in relation to demographic context was found when comparing respondents in cities and the countryside. In urban areas, (possibly due to the hiddenness of animal farming) both ideal pictures of happy farms (like in the song “Old MacDonald had a farm”), as well as very grim ideas of animal production, in terms of animal welfare and effects on the environment, were frequently brought forward, and urban respondents were found to opt for adapted meat consumption relatively more often than their rural counterparts. In rural settings, a self-pronounced “realistic” view of farming, accepting the system of rearing and slaughtering animals for food as a natural necessity, appeared to be the norm. This seems to be tied to the trend that in rural areas (in both countries) the interests of animals and nature taken into account in decision making were linked by respondents to practical value (“*To me, cows mean money.*” (planned conversation, Turkey, 15–30, rural)) more than to their intrinsic value, while in cities this was often the other way around. This difference between urban and rural inhabitants was the only difference that was spontaneously referred to by several respondents, both in rural: “*You know, those city folk, they have all these ideas in their heads about what farming is like, but they don’t know what it’s like. They’ve never even been on a farm!*” (planned conversation, Netherlands, male, 50+, rural) as well as in urban settings: “*Farmers have grown up with the ways of farming, they are dulled to how much the animals suffer.*” (planned conversation, Turkey, male, 30–50, urban). Still, “black swans” [89,90] were found in this encountered general pattern, as was the case with a farmer’s son in Turkey, who turned vegetarian after seeing what he referred to as his pet lamb being slaughtered at Eid.

#### 3.2.4. Gender

**Figure 2:**
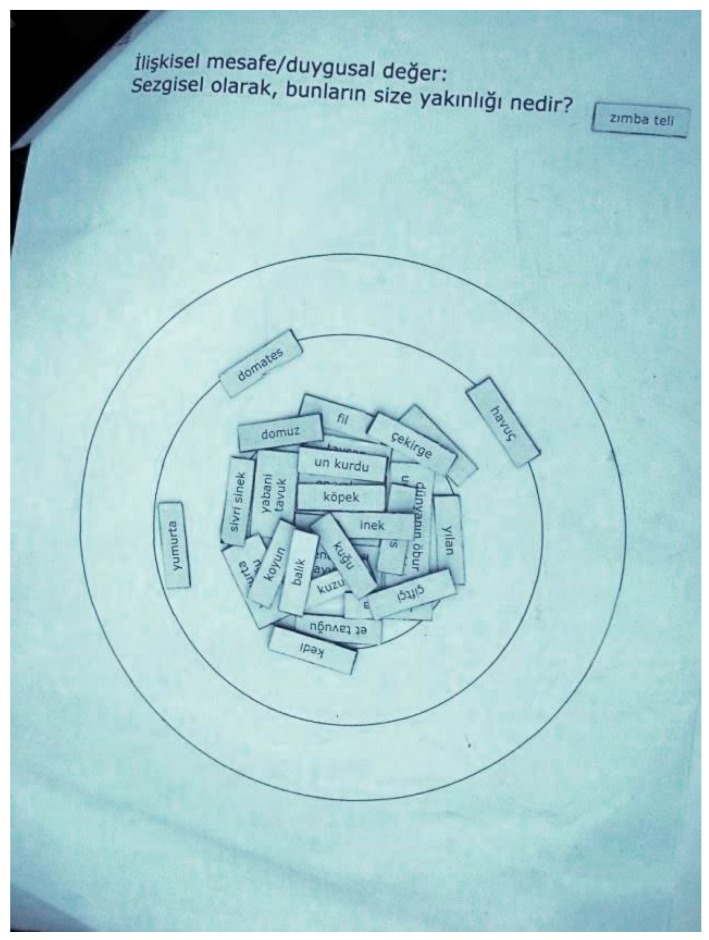
Circles of a respondent (Turkey, male, 15–30, urban) assigning equal importance to humans and other animals (the only cards positioned in the outer circles were *tomato*, *carrot*, and *egg*).

When comparing framing elements (including pronounced consumption behaviour), gender difference was not as salient in our research as would be expected based on the common stereotype that females are more empathic [18,91,92,93,94,95]: the expression of feeling empathy for farm animals was found relatively equally across male and female respondents in the research. An example of empathy in a (Turkish) male respondent is given in Figure 2, depicting not only the taking into consideration of animals, but the emotional equation of all living beings on the circles of relational distance.

#### 3.2.5. Summary

Summarising, single demographic contextual features did not offer as much insight into differences that showed up in the content of the framing as was expected based on literature. The qualitative research data does indicate several ties between mentioned framing elements and the researched case-study areas and population groups—the most salient pattern being the difference in framing of the (non-)acceptability of animal farming and meat consumption in urban vs rural geographic contexts. However, though in several opposite contexts topics were found that indeed were different, overall, recurring similarities and differences in composite framing were found far more saliently among various individual respondents in different contexts than in groups linked to the demographic contexts discussed above. In other words: symbolic convergence in lines of reasoning and composite framing—which could amply be found when comparing individual respondents, lacked a clear link to most of the single contextual factors that we expected to be of influence based on our literature research. (Note: as our article aims to provide our in-depth methodology, the elaborate overview of framing elements, and an account of the patterns that we encountered when looking at topics and composite frames in different contextual settings in Turkey and the Netherlands, a discussion of the symbolic convergence found among individual respondents falls beyond its scope.)

## 4. Discussion

### 4.1. On the Diversity of Topics Used

In scientific debates in the field of animal sciences, it has been customary to talk about the acceptability of animal farming and meat consumption in terms of “facts” and forgo opinions and feelings as “sentimental” [96]. Social acceptability of animal farming and meat consumption is a relatively new research area. Still, in the past decades, multiple consumer perception research projects on the issue have been initiated in the last few decades, though in particular focusing on animal welfare [1,3,7,8,9,10,11,12,13,15,16,17,23,24,25,26,27,28].

Our research adds to this body of knowledge, making clear that in everyday-life, talking about animal production and meat consumption is not a one-issue matter, and that constructing its (non-)acceptability does not consist of a rational display of facts but involves rich and multifaceted framing ingredients. Part of the framing may be about animals and their well-being, but referred-to topics (full list available at http://www.mdpi.com/2076-2615/8/2/17/s1) also include human health, nutritional value, meat taste and appearance, social relationships, religion, history, habits, consumption behaviours, global food supply, and environmental impact. Not only are facts hard to distinguish from opinions, our research indicates that feelings play a big role: the framing of farming is based on thoughts (the linguistic conceptualisations of our experiences, beliefs and opinions) as well as feelings (physical sensations, states/moods, and emotions).

The first implication following from these research results is that, to understand everyday-life framing of animal farming and meat consumption in its full complexity, focusing on factual knowledge is not enough. This is not to say that facts and rational concerns are not important: factual knowledge about climate change, for example, is what makes it an inescapable issue that value judgements or emotionally charged denial cannot counter. However, besides factual knowledge, the roles that unbacked convictions, values, and interests play in everyday-life framing cannot be discounted [65,66,68,69,97]. Furthermore, as feelings are what charges decisions with affect, they need to be acknowledged as equally—if not more—important reasons for certain framing to occur [97,98,99]. For scientists, philosophers, and societal stakeholders, this implies that trying to understand consumers by approaching the topic at a factual thinking level only, will provide a partial picture. For aspiring change-makers, this notion implies that fencing with facts without acknowledging and including people’s values, convictions, interests, and affective concerns, will likely be counterproductive, and make any meaningful interaction with consumers problematic.

Of course, the collected framing ingredients could have been organised in different ways, and codes and categories sometimes overlap; there are many ways to “slice a pie”. Nevertheless, distinguishing (sub-)categories and codes provided a useful way to systematically organise and analyse the pieces of information used in interaction to form frames. Moreover, we argue that, besides being a result of our inquiry into the framing of farming in the Netherlands and Turkey, the list of topics can be used as a tool for (self-)analysis, in which the accumulated codes and categories, aided by the accompanying eliciting questions, form a checklist to distinguish what thoughts and feelings are or may be of (conscious or unconscious) importance to ourselves and/or others when talking about farming, slaughter, and consumption of animals. Finally, though the list of topics is non-normative in itself, it could be used for having societal conversations about which topics do and should play a role when judging animal farming and meat consumption.

### 4.2. On the Complex Influence of Context

The second part of our research, in which we looked for patterns of symbolic convergence in relation to context, suggests that, besides some difference on the level of topics, general composite framings of animal farming and meat consumption related issues in Turkey do not differ from those in the Western European Netherlands, as much as would be expected based on literature. When looking at groups of respondents based on the other researched demographic characteristics, only the difference between framing in urban and rural areas clearly stood out: the research data suggest that generally romantic (painting a very positive picture of animal farming) as well as contrasting pessimistic views (often in relation with adapted consumption patterns such as vegetarianism) are uttered more in urban areas, while in rural areas, perceptions were relatively more rooted in “the way things are”, with an emphasis on the instrumental value of animals. Overall however, though in several opposite contexts some meaningful differences were found on the level of topics, patterns found in composite framing appeared far greater among individual respondents than patterns found between geographic contexts and population groups. Hence: symbolic convergence in lines of reasoning and composite framing—which was amply found when comparing individual respondents across the research—lacked a clear link to most of the single contextual factors that earlier research had made us expect to be of influence. Our framing analysis furthermore indicates that the way consumers talk about the issue also is linked to the personal situation at hand (daily life vs exceptional situations), and is linked to the parties that are taken into consideration by the individual consumer (the self, human beings, animals, the environment). One could continue looking for other distinct contextual factors that could be of influence on topic use, such as social network membership or political orientation. However, we argue that our research findings in this regard rather call for another approach, that involves taking into account the complexity of frame construction, happening in relation with multiple interdependent contextual features. Let us explain:

First of all, though categorised and coded as if separate, the ingredients used to frame animal farming and meat consumption in reality do not stand on their own, but are interrelated. To construct (non-)acceptability of animal farming and meat consumption in everyday-life, individual persons strategically select elements from the total of cognitions that is available to them in a specific situation, to suit goals they have in the interaction. The result is a complex combination of components, that as a whole, consist of more than the sum of its parts, because these mutually influence one another. In addition, framing elements can be flexible in meaning: negative or positive judgement may be added: “*That slaughter system for chickens, the running belt and machines taking their intestines out... Isn’t that awesome?! How far has mankind gotten in terms of technology!*” (pilot conversation, Turkey, male, 15–30, urban); a time frame or context may be interwoven: “*Red meat is healthy, because I feel it gives me strength to do demanding work, but in the long run it may be unhealthy. My father died of heart disease, so it runs in my family, and red meat is known to have negative effects on arteries.*” (planned conversation, Turkey, female, 50+, urban); and credibility may be played with: “*Some meat has a label. But it doesn’t matter what brand it has. Because I don’t trust those companies.*” (planned conversation, Netherlands, male, 50+, urban). In short: cognitions are not yet stories—they become so in combination and in interaction.

Frames as wholes, thus, are dynamic narratives, constructed strategically by tapping from topics available, each argument non-linearly adding to or subtracting from the acceptability of animal farming and meat consumption. Similarly, the context in which meaning is made, consists of manifold and interconnected features. The specific influence of the initially researched contexts (geographical location, gender, age groups etc.) already appeared weak when linking them to different framing elements. When looking at complex frames, the relation with context becomes even harder to identify. This is not to say that the framing of animal farming and meat consumption is not context dependent; nor that knowledge about the context in which frames are embedded does not add depth to its understanding. The conclusion is rather that *so many* cultural and personal contextual aspects play a role, that singular contextual features simply cannot sufficiently explain the way they are framed.

That the influence of context is complex, and the exact influence of individual factors is not easily isolatable, does not mean that there is no value in gathering knowledge about the context situation in which framing takes place. Framing and consumption behaviour are engrained in the cultural, esthetical, behavioural, and structural surroundings in which they take place [100,101,102,103]. Including a variety of (demographic but also more personal and situational) contexts and research subjects, moreover, adds depth to the understanding of the construction of each unique frame [77]. However, because contexts involve a multitude of features that mutually influence one another, and frames are complex non-linear composites of interrelated framing elements that are often flexible in meaning, looking at the influence of single contextual features and single framing elements (topics) provides but a partial picture. Future research into the framing of animal farming and meat consumption thus must consist of approaching the collected complex frames as wholes, embedded in their multifaceted social-cultural and personal contexts. When thus letting go of the focus on pre-determined demographic features and approaching the dataset as a whole, patterns may be distinguished in the way framing elements are combined that transcend these contexts.

### 4.3. Limitations and Extendibility of the Research

The research this article reports on focused on the analysis of (inter-)subjective human interpretations of the (non-)acceptability of animal farming and meat consumption, by analysing oral and written ways of everyday-life framing by consumers in the Netherlands and Turkey. Several limitations of the research project lie in the restrictions of this focus:

- The research focused on individual consumers and their framing. The interactions between consumers, as well as the frames of and interactions with other stakeholders, though very relevant for follow-up research, have not been part of this research.
- In the research, the exact definitions of farming systems (e.g., in terms of exact area available per animal, measures taken, kind of feed used, grazing/non-grazing, etc.) were only taken into account when respondents mentioned these. To better connect research on the social acceptability of animal farming such as ours with scientific debates in the field of animal sciences, a deeper focus on the (non-)acceptability of different systems is required.
- The positivist-empirical validation of the objective truthfulness of pronounced arguments regarding certain ways of animal farming and meat consumption and the effects they have on human health, the animals involved, or the environment—which is indispensable in the dialogue on animal farming and meat consumption related matters—has not been part of the current research, that purely regarded the interpretation hereof by consumers. Moreover, the research was focused only on pronounced behaviours, and did not check if actually performed behaviours matched these.
- The research was limited to specific urban and rural areas in the Netherlands and Turkey.
- Quantitative confirmations of the influence of demographic contextual factors or demographic distribution of the use of specific clusters of reasoning and behaviour in framing have not been carried out within the timeframe of this study.

Though it was tailored at increasing understanding about the everyday-life framing of animal farming and meat consumption in the specific case-study areas in the Netherlands and Turkey, we argue that our research comprises enough qualitative detail (depth) and ranges over enough contexts and people (scope) to be able to extend the research findings to at least similar people in similar contexts [104]. With regard to the distinguished list of topics, we would also argue that generalisability of the results beyond the European sphere is probable, though more research is needed to confirm that. Details in content, such as exact topics (norms, values, interests, etc.) referred to, are likely to differ—especially in areas with very different food cultures such as Africa, South-East Asia, or the Arctic. For the analytical tool to be used to understand other actors and other contexts, it would first need to be adapted and tested. The results, however, can arguably be used to initiate dialogues in the European sphere as well as other parts of the world, with the provision that they are based on research in Dutch and Turkish contexts.

## Figures and Tables

**Table 1:** Items used in the visualization exercise (in no particular order).

me (placed in the centre of the circles)	staple (placed outside the circles)	someone on the other side of the world	my pet (if existent)
tomato	sheep	lamb	grasshopper
fish	a farmer	rabbit	broiler chicken
cow	dolphin	mosquito	wild chicken
pig	Swan	snake	my best friend
dog	carrot	laying hen	elephant
frog	flour worm	horse	cat

**Table 2:** Consumption behaviours and the way people performing it are commonly referred to.

	Red meat	Poultry	Fish	Eggs	Dairy	Honey	Grains	Roots	Vegetables	Fruits	Nuts and seeds	Beans
**Omnivore** Eats animals, animal products, as well as plants	**✓**	**✓**	**✓**	**✓**	**✓**	**✓**	**✓**	**✓**	**✓**	**✓**	**✓**	**✓**
**Carnivore** Eats mostly meat	**✓**	**✓**	**✓**									
**Vegetarian** Refrains from eating meat or fish, does eat eggs and dairy				**✓**	**✓**	**✓**	**✓**	**✓**	**✓**	**✓**	**✓**	**✓**
**Vegan** Refrains from eating and using any animal products							**✓**	**✓**	**✓**	**✓**	**✓**	**✓**
**Lacto vegetarian** Refrains from eating meat, fish and eggs, does eat dairy					**✓**	**✓**	**✓**	**✓**	**✓**	**✓**	**✓**	**✓**
**Ovo vegetarian** Refrains from eating meat and dairy, does eat eggs				**✓**		**✓**	**✓**	**✓**	**✓**	**✓**	**✓**	**✓**
**Pescotarian** Refrains from meat and poultry, does eat fish			**✓**	**✓**	**✓**	**✓**	**✓**	**✓**	**✓**	**✓**	**✓**	**✓**
**Pollotarian** Refrains from red meat and fish, does eat poultry		**✓**		**✓**	**✓**	**✓**	**✓**	**✓**	**✓**	**✓**	**✓**	**✓**
**Fruitarian** Refrains from eating “living” plants (roots), does eat their products (fruits, nuts, beans)									**✓**	**✓**	**✓**	**✓**
**Flexitarian** Consciously eats less meat	**✓**	**✓**	**✓**	**✓**	**✓**	**✓**	**✓**	**✓**	**✓**	**✓**	**✓**	**✓**

## Supplementary Materials

The following are available online at http://www.mdpi.com/2076-2615/8/2/17/s1: List S1: Categories and codes: topics used to talk about animal farming and meat consumption.

## Appendix A

**Table A1:** Overview of the demographic characteristics of the 50 respondents that were selected for the planned semi-structured in-depth conversations.

	Country	Main Location	Link with Animal Farming	Gender	Age Group	Education Level	Occupation Type	Income Level (Able to Buy Desired Food?)	Pronounced Protein Consumption
1	NL	Urban	No	M	15–30	Student	High school student, not yet working	Sufficient	Regular meat eater (Halal)
2	NL	Urban	No	M	15–30	Middle	Sales, youth work	Sufficient	Regular meat eater (Halal)
3	NL	Urban	Half	M	30–50	High	NGO office work	Sufficient	Compromise (only from ‘good sources’)
4	NL	Urban	No	M	50+	Low	Retired labourer	Sufficient	Regular meat eater
5	NL	Urban	No	F	15–30	Low	Cleaning	Sufficient	Regular meat eater
6	NL	Urban	No	F	30–50	High	Journalism, writing	Sufficient	Compromise (less meat and only organic)
7	NL	Urban	No	F	30–50	High	Horticulture	Minimal	Compromise (less meat and only from ‘good sources’)
8	NL	Urban	No	F	30–50	High	Aquatic science	Sufficient	Compromise, mostly vegetarian
9	NL	Urban	No	F	50+	High	High school teaching	Sufficient	Compromise (just fish)
10	NL	Mixed	No	M	15–30	Middle	Intern, communications	Minimal	Regular meat eater/Compromise (less meat)
11	NL	Mixed	No	M	15–30	High	Volunteer coordinator	Sufficient	Vegetarian
12	NL	Mixed	Half	M	50+	Low	Jobless and homeless	None	Meat eater (no compromise)
13	NL	Mixed	No	M	50+	High	Lawyer	Sufficient	Vegetarian
14	NL	Mixed	No	F	15–30	High	Campaigner	Minimal	Vegetarian
15	NL	Mixed	No	F	30–50	High	Secretary	Sufficient	Compromise
16	NL	Mixed	Half	F	30–50	High	Computer engineer	Sufficient	Regular meat eater
17	NL	Mixed	No	F	30–50	High	Food scientist	Sufficient	Regular meat eater/Compromise
18	NL	Mixed	No	F	50+	High	Creative therapist	Sufficient	Vegetarian/Compromise
19	NL	Rural	No	M	30–50	Middle	Mechanic and fireman	Sufficient	Regular meat eater
20	NL	Rural	Yes	M	30–50	Middle	Organic farmer	Sufficient	Compromise (only organic)
21	NL	Rural	Yes	M	50+	Low	Dairy farmer	Sufficient	Regular meat eater
22	NL	Rural	Yes	F	50+	Low	Farm worker	Sufficient	Regular meat eater
23	NL	Rural	Yes	F	50+	Low	Farmer’s wife	Sufficient	Regular meat eater
24	NL	Rural	No	F	50+	Middle	Forest conservationist	Sufficient	Vegetarian/Compromise (sometimes fish)
25	NL	Urban	Half	F	30–50	High	Researcher	Sufficient	Flexible vegan
26	TR	Urban	No	M	15–30	High	Student, film director	Sufficient	Compromise
27	TR	Urban	No	M	15–30	High	Doctor’s assistant	Sufficient	Regular meat eater
28	TR	Urban	No	M	30–50	High	Captain	Sufficient	Compromise (less meat, only chicken and fish)
29	TR	Urban	Yes	M	30–50	Low	Butcher	Sufficient	Regular meat eater/compromise
30	TR	Urban	No	M	30–50	High	NGO campaigner and radio host	Sufficient	Vegetarian
31	TR	Urban	No	M	50+	High	Retired leather shop owner	Sufficient	Vegetarian/Compromise (only wild animals)
32	TR	Urban	No	F	15–30	Stud.	High school student	Sufficient	Compromise (trying out vegetarianism)
33	TR	Urban	No	F	15–30	High	Social scientist	Sufficient	Vegetarian, thinking about going back to eating meat
34	TR	Urban	No	F	30–50	High	Tourism guide	Sufficient	Compromise (was vegetarian, eats meat only on the job)
35	TR	Urban	No	F	50+	Middle	Secretary	Sufficient	Compromise (no red meat, less meat)
36	TR	Urban	No	F	30–50	Middle	Restaurant co-owner	Sufficient	Regular meat eater
37	TR	Urban	No	F	30–50	High	Nurse	Sufficient	Regular meat eater
38	TR	Urban	No	F	30–50	High	Mathematics teacher	Sufficient	Regular meat eater
39	TR	Urban	No	F	30–50	High	Translator	Sufficient	Vegetarian
40	TR	Urban	No	F	30–50	Low	Mother, photographer, stray animal welfare worker	Minimal	Vegetarian/Compromise (feeds meat to her animals, sometimes eats a bit)
41	TR	Urban	No	F	50+	High	Banker, hotel owner	Sufficient	Regular meat eater
42	TR	Mixed	No	M	15–30	Low	Janitor	Minimal	Compromise
43	TR	Mixed	No	M	15–30	High	Food scientist, factory owner	Sufficient	Regular meat eater
44	TR	Mixed	No	M	50+	Middle	Accountant	Sufficient	Regular meat eater
45	TR	Mixed	No	M	50+	High	Imam	Sufficient	Regular meat eater
46	TR	Mixed	No	F	15–30	High	Ecologist	Sufficient	Vegetarian/ Vegan
47	TR	Rural	Yes	M	15–30	Student	Farmer’s son	Minimal	Regular meat eater
48	TR	Rural	Yes	M	30–50	Middle	Horse therapist and sheep farmer	Sufficient	Regular meat eater
49	TR	Rural	Yes	M	30–50	Low	Part-time farmer and restaurant manager	Sufficient	Regular meat eater
50	TR	Rural	Yes	F	50+	Low	Farmer	Very low	Regular meat eater
