# Supplementary material for: Exploring the Framing of Animal Farming and Meat Consumption: On the Diversity of Topics Used and Qualitative Patterns in Selected Demographic Contexts
Source: Animals (Basel). 2018 Jan 24;8(2):17. doi: 10.3390/ani8020017 (PMC5836025; doi:10.3390/ani8020017)
Supplement: Supplementary file 1 [file animals-08-00017-s001.pdf]

List SI: Categories and codes - topics used to talk about animal farming and meat consumption

In this overview, we list the six main categories of framing *behaviours, values, norms, feelings, interests, and knowledge and convictions*, and associated sub-categories and codes that we have distilled from the data of our research into the framing of animal farming and meat consumption in the Netherlands and Turkey. Together, they provide an overview of the various topics used to talk about the farming, slaughter and consumption of animals in everyday-life. With each category, we have listed questions that can be used to probe for topics that play a role.

Ingredient 1: Behaviours

When framing animal farming and meat consumption, one of the most important things people refer to are their *behaviours*. We define behaviours as pronounced personal past and present actions, including habits and exceptions. Behaviours thus are references to what *is (or was) done*.

Questions that can be used to elicit behaviours in conversations are:

- *What do you generally do? How do you do it?*
- *Did/do you always do it in the same way?*
- *When did/do you do it differently? How?*
- *What were exceptional behaviours?*

1.1 Personal consumption pattern

A first and central topic within the category of behaviours that people refer to in their everyday framing regarding the farming, slaughter and consumption of animals, is their personal consumption pattern: the extent to which meat is eaten. This, it turns out, cannot not simply be categorised as 'meat eating' vs 'vegetarianism'. In my research, most self-named meat eaters still refrained from eating certain species or parts of the animal – 'all-meat' consumers are not all that common. In Turkey for example, meat should be of Halal origin. Furthermore, there were many 'in-between' behaviours, for example eating less meat or only meat from organic origin. Finally, there were people refraining not only from meat, but also other animal products ('veganism'). In selected cases, additional rituals and habits were mentioned as part of the consumption pattern, such as praying before eating or not throwing meat away.

Sub-categories and codes:

- Eating meat
    - Anything goes
    - Regularly available meat
    - Only meat from certain species
    - Only certain parts of the animal
    - Only cooked meat
    - Only meat from certain origin/with certain label
- Refraining from animal products
    - Vegetarianism
    - Veganism
    - Fruitarianism
- Flexitarianism (eating meat in moderation)
    - Quantity per instance
    - Frequency
- Additional rituals and habits regarding consumption
    - Paying respect to the food
    - Not wasting food

- Eating in specific spaces or in specific company
- Mindfulness in eating

1.2 Personal practices regarding animal farming and slaughter

Several consumers, predominantly in rural areas, have personal experience with animal farming and slaughter due to their profession (e.g. farmer, agricultural contractor, butcher) or because they have killed an animal on occasion (e.g. for the Sacrifice feast, or for personal consumption). In their pronounced behaviours the distinction was made between regular and adapted farming and slaughter practices.

Sub-categories and codes:

- Regular farming and slaughter practices
  - Small holder type farming
  - Industrial type farming
  - Industrial type slaughter
- Adapted farming and slaughter practices
  - Free-range type farming
  - Organic farming
  - Biodynamic farming
  - Hobby farming
  - Ritual slaughter
  - Hand slaughter
  - Other rituals adopted regarding farming and/or slaughter
  - Feeding a non-herbivorous pet

1.3 Other behaviour related to animal farming and meat consumption

Next to practiced consumption behaviour or farming/slaughter style, several other activities are mentioned as relevant to the (non-)acceptability of animal farming and meat consumption, such as information oriented activities, financial activities and political activities.

Sub-categories and codes:

- Information oriented activities
  - Having observed information (/source)
  - Farm and slaughterhouse visit
  - Active knowledge pursuit
  - Transmission of information, awareness raising
- Financial activities
  - Spending money on consumption pattern
  - Saving money on consumption pattern
  - Investing in or earning money from animal production system
- Political activities
  - Voting
  - Social movement membership

1.4 Reflecting on behaviour

Besides mentioning current usually performed behaviours, some people also *reflect* on their behaviours - for example by mentioning behaviours that are exceptional in some way or revealing insights about the consistency of their behaviour or changes in history. Moreover, on an even deeper level, some people make a real effort to reflect on the habitualness or consciousness of the process of decision making.

Sub-categories and codes

- Exceptions to common personal behaviour

- How I eat/ate differently out of hunger
  - How I eat/ate differently out of politeness / regard for others
  - How I eat/ate differently at special occasions (feasts, special gatherings)
  - How I eat/ate differently when I was feeling ill
  - How I eat/ate differently out of curiosity
  - How I eat/ate differently out of budgetary reasons
  - How I eat/ate differently out of a craving
  - How I eat/ate differently out of occupation with other things (business, laziness, ease)
  - The way I treat(ed) some animals differently during farming
  - The way I treat(ed) some animals differently when killing them
- Consistency/flexibility and change in behaviour
  - How consistent I am in my personal behaviours
  - The contexts in which personal behaviours differ
  - The way my common personal behaviours changed over time
- Consciousness and comprehensiveness of decision making
  - How conscious I am when deciding on personal behaviours and exceptions
  - How comprehensive I am (inclusive of all my thoughts and feelings)
  - How much personal behaviours and exceptions are habitual
  - Whether a possible change has become a new habit or still requires consciousness

Ingredient 2: Values

In framing the (non-)acceptability of animal farming and meat consumption, individual people inevitably make use of the values they hold – values that may or may not coincide with mainstream values held in the society around them. Values are often implicit, but after asking repeated *"why is that important to you?"* type questions, people also mention them explicitly. We define values as rational concerns, conceptualisations about what and whom is considered important and to what extent (which includes valued ways of decision making).

Questions that can be used to elicit values in conversations are:

- *What is important to you? Who is important? How important? And what and who is not?*
- *What and who do you rationally take into account when making decisions?*
- *What is the best way to come to a decision?*
- *What kind of person do you want to be?*
- *Why is that important to you? How do you decide that?*

2.1 Attribution of value and consideration of interests

When talking about the (non-)acceptability of animal farming and meat consumption, the attribution of value and consideration inevitably comes up: *what or whom* to take into account and to what degree. Assigning value happens in two ways, namely the attribution of *practical value* (the practical importance or use something or someone has to ourselves or other humans – in philosophy referred to as *instrumental value*) and the attribution of *rational intrinsic value* (the value something or someone is thought to have in and of its own, regardless of its use).

Related to these values (much overlapping – but distinctly important for the framing of animal farming and meat consumption and therefor a separate code category) is the *consideration of interests* (i.e. valuing a being or entity to the extent that its interests must be given consideration in decision making). Even though certain beings were assigned intrinsic value to, in the daily framing of animal farming and meat consumption this does not necessarily mean that their interests were considered important enough to take into account in the actual decision-making process. In my research, the interests that were said to be considered were at minimum the respondents themselves and human beings that are close by (relationally, temporally and/or spatially). In some cases, this stretched via the interests of all human beings, to all living beings and future generations. Only to a limited extent the interests of the earth/environment were really considered (more often through the practical

value of environmental protection for the interests of us human beings). However, even if interests of for example animals were considered important enough to take into consideration, they were often pronounced to be of *less* importance compared to the interests of for example our loved ones.

Sub-categories and codes:

- Practical value
  - Meat
  - A clean environment
  - Animals (resource/capital)
  - The practice of farming and slaughter of animals
  - The trade in animal-based products
- Rational intrinsic value
  - Human beings
  - Animals
  - Plants
  - The earth/the environment
  - Future generations
- Consideration of interests
  - The self
  - Loved ones (human and non-human)
  - Group members
  - Human beings in general (whether close by or on the other side of the world)
  - All living beings (with a possible order of species preference, but including farmed animals)
  - The environment, earth or biosphere
  - Future generations

2.2 Personal values held

Regarding *what* is found important, there are several rational concerns that relate directly to farming, slaughtering and consumption of animals, such as basic survival, profit and saving money, food safety, food quality, protecting health, food culture, farmer livelihood, ensuring the supply of sufficient food to feed the word, and moreover in a fair and fairly distributed way. Regarding the animals themselves, their health, welfare, and rights, the ability to perform natural behaviour, the no-harm principle, sometimes extended to the no-kill principle, were values that were referred to. Finally, environmental protection and sustainability were mentioned as being important rational concerns.

Next to this, more general personal values are mentioned as relevant when framing animal farming and meat consumption, that relate to the kind of person someone wants to be, such as being objective, consistent or successful, being realistic, being compassionate, tolerant or polite, being intuitive, virtuous, responsible, informed or a change agent.

Sub-categories and codes:

- Rational concerns regarding animal farming and meat consumption
  - Preserving life, survival
  - Profit/saving money
  - Food safety
  - Food quality
  - Health
  - Food culture
  - Farmer livelihood
  - Farming culture
  - World food supply
  - Fair trade and distribution of food

- Animal health
    - Animal welfare
    - Naturalness, ability to perform the natural behaviour
    - Quality of life according to the animal’s function and capability
    - Animal rights
    - No-harm principle
    - No-kill principle
    - Waste reduction
    - Environmental protection
    - Sustainability, safeguarding future generations
    - Protection of depth of consciousness
- Other relevant personal values
    - Objectivity, rationality, impartiality
    - Consistency, reliability
    - Successfulness
    - Being in touch with natural instincts, raw
    - Realism
    - Autonomy, freedom
    - Being in touch with emotions
    - Compassion, empathy, care
    - Being protective
    - Belonging
    - Tolerance
    - Non-interference
    - Politeness
    - Being in touch with intuition, wisdom
    - Virtue, being a good person
    - Purity
    - Justice, equality, fairness
    - Responsibility, accountability
    - Being aware and informed, inquisitive
    - Contributing to a better world
    - Feminism
    - Holistic approach

2.3 Valued ways of decision making

Knowing whose interests and what values are important, does not suffice to arrive at a deliberated conclusion on what one’s course of action should be. When framing animal farming and meat consumption, people therefore also refer to the ways of decision making they value. These are often deliberate ways of decision making, based on the ethical ideals of weighing positive and negative outcomes of a decision (consequentialism), living by duties and rights based on principles (deontology), referring to the role of humans or the kind of person one wants to be (virtue ethics), and pursuing inquiry into the validity of moral criteria (pragmatism).[1]

A different way of arriving at a decision that was pronounced to be of value is intuitive deciding. This way of decision making involves a description of a combination of rational reasoning with trusting of a 'gut feeling', 'inner knowing' or even a 'tapping into the field of knowing wider than ourselves' when making a new decision situation. This does not deny the importance of rational decision making (hence it is mentioned as a rationally valued way of decision making), but is more comprehensive: it includes thoughts as well as feelings, that are both within and outside of conscious awareness (thus not to be confused with purely heuristic decision making).

---

[1] See e.g. VanDeVeer, D., & Pierce, C. (1986). *People, Penguins, and Plastic Trees: Basic Issues in Environmental Ethics*. Belmont: Wadsworth.; Zweers, W. (1995). *Participeren aan de natuur: ontwerp voor een ecologisering van het wereldbeeld*. Utrecht: Van Arkel. and Van den Berg, F. (2013). *Veganism: a moral imperative*. Universiteit Utrecht (poster).

Besides referring to valued ways of decision making, some ways of deciding are explicitly mentioned as being inferior (not-valued), such as decision making lacking rational thought, solely based on feelings. Conversely, purely rational weighing of factual pros and cons has in cases also been criticised as lacking empathy and not being sensitive enough to ethical and aesthetical arguments. Non-conscious decision making, based solely on heuristic habits or instincts, furthermore was seen as inferior.

Sub-categories and codes:

- Deliberate decision making
    - Weighing positive and negative outcomes of a decision (consequentialism)
    - Living by duties (and sometimes rights) based on principles (deontology)
    - Referring to the role of humans, the kind of person one wants to be (virtue ethics)
    - Pursuing inquiry into the validity of moral criteria (pragmatism)
- Intuitive decision making
- Negatively valued ways of decision making
    - Only feeling-based deciding
    - Only rational deciding
    - Non-conscious, instinctive, impulsive and/or habitual deciding
    - Biased decision making

Ingredient 3: Norms

An important ingredient of the framing of animal farming and meat consumption are norms. As the name gives away, norms are normative, prescriptive in nature. As explained in section 2.1, in the original model of the frame-of-reference they were defined as 'translation of values into rules of conduct'. Norms indeed are importantly derived from values, in particular the consideration of interests and the valued ways of decision making. In practice however, these rules of conduct importantly get influenced by other frame-of-reference elements, such as knowledge & convictions, interests, and feelings. We thus define norms as simply what is brought forward that *should be done*: ideal rules of conduct imposed on the self - and possibly others.

In coding content, norms are very similar to the behaviours mentioned in the context of animal farming and meat consumption. However, in contrast to pronounced behaviours, that are statements about what really happens, norms are ideal behaviours, meaning they do not always get executed in real life. In everyday-life framing of animal farming and meat consumption, norms form the ingredient that defines theoretical (non-) acceptability, the other ingredients are used to underpin and develop these rules of conduct, or justify their non-execution. Primarily, these norms are rules that apply to oneself, and are mentioned as such, though they are also importantly brought forward to prescribe expected behaviours to others.

Mentions of norms that are alive in society and descriptions of what is considered the norm ('normal') in certain (sub-)cultures, fall under ingredient 6: knowledge & convictions.

Questions that can be used to elicit norms in conversations are:

- *How does what you find important translate into action?*
- *How would you like to act?*
- *What do you think should be done?*
- *Who is responsible for performing what should be done?*
- *What is acceptable and what not?*
- *What are the minimum requirements? What should be there?*
- *Can you describe your ideal picture?*

3.1 Norms regarding food

Depending on one's knowledge and convictions, one's physical state, one's values and one's ideas on whose interests should to be taken into consideration when deciding on the (non-)acceptability of animal farming and meat consumption, norms are formulated regarding food. These range from basic food requirements such as food safety and health, to norms about refraining from (certain parts of) animal meat due to religious convictions or consideration of the interests of animals and the environment. Also, in this regard mentions are made on how one should approach food that's on the plate.

Sub-categories and codes:

- Basic food requirements
  - Food safety, health
  - Food security
- Refraining from animal products
  - Vegetarianism
  - Veganism
  - Fruitarianism
- Flexitarianism (eating meat in moderation)
  - Quantity per instance
  - Frequency
- Eating meat
  - Anything goes
  - Regular meat
  - Only meat from certain species
  - Only certain parts of the animal
  - Only cooked meat
  - Only meat from certain origin/with certain label
- Additional norms regarding consumption
  - Paying respect to the food
  - Not wasting food
  - Eating in certain spaces or in specific company
  - Eating mindfully
  - Pet feeding norms

3.2 Norms regarding animal farming and slaughter

An important part of the framing of animal farming and meat consumption are of course norms regarding actual farming and slaughter practices. These regard both norms imposed on oneself as farmers or slaughters (forming the so called 'licence to produce'), as well as those imposed on others who farm and slaughter animals for a living, and range from basic legal standards to far reaching additional standards and the abolishment of farming and slaughter altogether.

Sub-categories and codes:

- Basic standards for farming and slaughter
  - Small holder type farming
  - Industrial type farming
  - Industrial type slaughter
- Additional standards for farming and slaughter
  - Free-range type farming
  - Organic farming
  - Biodynamic farming
  - Hobby farming
  - Ritual slaughter
  - Hand slaughter

    - Other rituals adopted regarding farming and/or slaughter
- Abolishment of farming
- Abolishment of slaughter

3.3 Other norms related to animal farming and meat consumption

Next to rules on consumption behaviour or farming and slaughter styles, other guidelines for action that were brought forward regarded management of finances, political action, information seeking, and valued ways of decision making.

Sub-categories and codes:

- Management of finances
    - Spending money on consumption pattern
    - Saving money on consumption pattern
    - Investing in or earning money from animal production system
- Political action
    - Voting
    - Social movement membership
    - Lobbying for legislation, targeted subsidies and taxes
    - Awareness raising
- Objective information seeking
- Norms for making decisions
    - Decide rationally
    - Decide intuitively
    - Avoid negatively valued ways of decision making

3.4 Parties that norms are assigned to

The parties that are held responsible to perform the rules of conduct, form a last and essential sub-category of norms. The parties included here describe whom the abovementioned behavioural norms are imposed on.

Sub-category and codes:

- Attribution of responsibility
    - Myself
    - Consumers
    - Producers
    - Government
    - Retail
    - The system/the market
    - Everyone

Ingredient 4: Feelings

An important ingredient in the construction (non-)acceptability of animal farming and meat consumption are feelings people state they have. Whereas values are *rational* concerns about what and whom is considered important and to what extent, feelings are *affective* concerns. As we define them, feelings comprise of three, much interrelated, categories: physical sensations (images, smells, sounds, tastes and tactile input, leading to a sense of the outside world as well as one's inner world), states (moods and other temporary but relatively sustained felt modes of being) and emotions (subjective positive, negative or neutral associations with experiences or thoughts). Pur sang, feelings are non-linguistic; however, they are an important element of verbal framing, for people continuously *refer* to feelings. The linguistic representation of feelings is often accompanied by gestures, facial expressions and an emotionally charged tone of voice.

Questions that can be used to elicit feelings in conversations are:

- *How does it make you feel?*
- *Who and what feels important, close to you?*
- *Where in your body do you feel that?*
- *What do you sense physically?*
- *What state or mood are you in?*
- *What emotions come up?*
- *What touches you? What do you dislike? What do you like?*

4.1 Felt relationship

Felt relationship delineates the others that are cared for, that they felt emotionally or relationally close to. This ranged from human beings that are close by, to all human beings, to all living beings, the earth, and future generations (cf. the rational consideration of interests).

Sub-categories and codes:

- Affective concerns/emotional/relational value
    - The self
    - Loved ones (human and non-human)
    - Group members
    - Human beings in general (whether close by or on the other side of the world)
    - All living beings (with a possible order of species preference, but including farmed animals)
    - The environment, earth or biosphere
    - Future generations

4.2 Feelings about consumption of meat, meat types and alternatives

An important category in which physical sensations, felt states and emotions are mentioned, is the consumption of meat and alternatives to meat. These feelings are declared to play a role at both real as well as imaginary consumption, so during actual eating and during thinking about eating, for example at the obtainment of food. Falling in this category are the personal physical food experience, with topics such as taste, smell and structure; states related to food consumption such as hungry, satisfied, sick or vital; and emotions regarding food consumption such as joy, gratitude, pride, and disgust.

Sub-categories and codes:

- Personal physical food experience
    - Taste
    - Smell
    - Structure
    - Appearance
    - Rumbling stomach
    - Satisfied stomach
    - Watering mouth
    - Aching/upset stomach
- States related to food consumption
    - Hungry
    - Satisfied
    - Weak, lacking in energy
    - Sick
    - Comfortable
    - Vital, healthy, energetic, excited
    - Strong, powerful
    - Calm
    - Agitated

- Cheerful, good mood
- Depressed
- Conscious, mindful
- Unconscious, habitual, thoughtless
- Instinctive, wild, rough

- Emotions regarding food consumption
  - Joy, happiness
  - Gratitude
  - Pride
  - Nostalgia
  - Disgust
  - Frustration, stress
  - Guilt
  - Anger
  - Indignation
  - Sadness
  - Neutrality/indifference

4.3 Feelings about animal farming and slaughter

Besides the consumption of meat and non-meat products, feelings are expressed specifically related to the practice of rearing and slaughter of consumption animals. Again, this both pertains to the feelings that come up during the actual animal production process, as well as feelings that come up during memories and imaginations of oneself and/or others partaking in it. It includes physical experiences such as smell and appearance; states such as strong, instinctive, cheerful, agitated, and depressed; and emotions such as joy, pride, love, sadness, anger and guilt.

Sub-categories and codes:

- Physical experience of farming and slaughter practices
  - Smell
  - Touch
  - Appearance, sight (whether real or on video)
  - Itching fingers
  - Upset stomach
- States related to farming and slaughter practices
  - Vital, strong, powerful
  - Calm
  - Excited
  - Agitated
  - Cheerful, good mood
  - Depressed
  - Conscious, mindful
  - Unconscious, habitual, thoughtless
  - Instinctive, wild, rough
  - Empathic, respectful
  - Selfish
- Emotions regarding farming and slaughter practices
  - Joy
  - Gratitude
  - Pride
  - Love
  - Nostalgia
  - Frustration, stress
  - Indignation

- Anger
- Sadness
- Disgust
- Guilt
- Neutrality/indifference

4.4 Other relevant feelings

Besides feelings directly related to the acts of rearing, slaughtering and consuming animals, other feelings are in play when framing animal farming and meat consumption. These regard first of all feelings *about* the values, norms, interests, knowledge & convictions they themselves hold, behavioural decisions they make, and feelings they have. Also relevant are feelings about the perceived values, norms, interests, feelings, behavioural decisions and knowledge & convictions of others, as well as feeling about the way society is organised. A final, but rather important category of feelings regards ambivalence and dissonance: for people report having various feelings about discrepancies between performed behaviour, drives, and cognitions (of oneself or perceived in others).

Sub-categories and codes:

- Feelings about oneself
    - Own values
    - Own norms
    - Own interests
    - Own knowledge & convictions
    - Own behavioural decisions
    - Own feelings
- Feelings about others
    - Others' values
    - Others' norms
    - Others' interests
    - Others' knowledge & convictions
    - Others' behavioural decisions
- Feelings about the way society is organised (more in general than specifically regarding consumption options and farming and slaughter styles)
- Feelings about ambivalence and dissonance (ambiguity, contradiction, conflict)
    - Neutrality/indifference
    - Doubt
    - Uncomfortable tension
    - Acceptance
    - Feeling good by virtue of the acceptance of the tension

Ingredient 5: Interests

The recognition, formulation and defending of interests is an important part of the framing of animal farming and meat consumption. The coding category *interests* regards one's personal interests: recognised stakes and goals that inner drives (consciously or unconsciously) motivate us to strive for, both material (physical, economic) as well as immaterial (social, moral, aesthetical). The framing content coded under interests thus includes both the stakes people recognise they have in a certain matter, as well as talk about the inner urges or drives that are noticed, defined in terms of the goals they motivate us to strive for. Of course, there are multiple ways to categorize these. One could for example distinguish groups based on e.g. short-term and long-term goals or unambiguous needs versus mere 'would-likes'. Here we have chosen a categorisation based on the distinction between an individual's moral convictions, their desire to take care of one's exterior physique, their need to belong in groups and otherwise manage their relationship with others, and their aiming to live in this world. Perceived interests of others are dealt with in Ingredient 6: Knowledge and convictions.

Questions that can be used to elicit interests in conversations are:

- *What do you want?*
- *What do you feel urged to do?*
- *What would be nice to have or do?*
- *What do you really need?*
- *What do you want to achieve?*
- *What are your goals and intentions?*
- *What are the stakes involved for you?*
- *How would this behaviour benefit or impair you?*
- *What do you want to avoid?*

5.1 Living according to one's moral convictions

The first category of interests that people referred to regard wanting to live according to one's personal moral convictions. The first sub-category deals with moral conviction of a general and specific nature that are applied to the issue of animal farming and meat consumption. The second sub-category is a special one, that regards *decisions* that are (to be) made in the past, present or future.

Sub-categories and codes:

- Wanting to live according to moral convictions *(see also ingredient 2: values)*
  - Being well-informed
  - Being tolerant
  - Being realistic
  - Being autonomous
  - Being in touch with natural instincts
  - Being consistent
  - Contributing to aesthetics
  - Being compassionate, empathic, caring
  - Protecting human well-being
  - Protecting animal well-being
  - Protecting the environment
  - Living sustainably, safeguarding future generations
- Wanting to come to one's decisions in a fair and high quality manner *(see also ingredient 2.3)*
  - Rational decision making
  - Intuitive decision making
  - Avoid negatively valued ways of decision making

5.2 Taking care of one's physique

The second set of interests focuses on safeguarding physical and emotional needs and preferences, or in other words: taking care of one's physique. The second sub-category here again is of a special nature, regarding the fairness and quality of the *decision*, rather than consisting of animal farming and meat consumption related topics.

Sub-categories and codes:

- Wanting to take care of one's physique *(see also ingredient 4: feelings)*
  - Survival
  - Health
  - Physical well-being
    - Food experience
    - Satisfaction of the senses
    - Comfort
  - Emotional well-being
  - Labour satisfaction

    - Habit continuation
- Wanting to feel good about one's decisions (past, present or future)
    - Dealing with uncomfortable feelings regarding dissonance

5.3 Managing one's relationships

The third category of interests relates to various aspects of managing one's relationships with others (that are found of value: see ingredient 2.1).

Sub-categories and codes:

- Wanting to manage one's relationships (See also ingredient 4.1: felt relationship)
    - Connection with others
    - Belonging
    - Social status management
    - Face-saving
    - Food culture
    - Farming culture
    - Cultural identity (in a broader sense)
    - Caring for/helping others (i.e. empathizing with and helping them attain their goals)

5.4 Living in the world

The last category of interests that people refer to, regards dealing with (perceived or real) physical limitations, or in other words: managing to live in the world. This type of interests can be approached in two ways: by trying to adapt to the structural reality or by trying to change it.

Sub-categories and codes:

- Wanting to live in the world (see also ingredient 6: knowledge & convictions)
    - Adjusting to the current economic reality
        - Budgeting existing money
        - Generating new financial resources
    - Complying with existing policy and legislation
    - Adjusting to the accessibility of information
    - Adjusting to the availability of alternatives
    - Trying to change the economic/legal/institutional/socio-cultural systems

Ingredient 6: Knowledge & convictions

Last but not least, a lot of knowledge & convictions are brought forward with regards to animal farming and meat consumption related issues: opinions about the way things are, about (self-)efficacy and the effects certain situations will have, associations and assumptions about what is true; including the perceived behaviours, values, norms, feelings, interests, and knowledge & convictions of others.

As already touched upon in section 2.1 of our article, the distinction between empirically observed facts and untested convictions is not at all that easy to make - which was confirmed during the gathering and analysing of data. For example, the sentence *"My neighbour is a vegetarian"* does not unmistakably show whether this is empirically observed knowledge, reported speech, or an assumption. While there arguably is a difference between for example *"Science has proven that sows suffer in birth cages"* (reported speech, pointing towards factual information) and *"I believe that sows suffer in birth cages"* (direct speech, using 'I believe', pointing at this being a conviction), the *topics* referred to are the same (in this example: the impact of housing, measures and treatment on animals). Because of this overlap and the uncertainty in distinguishing fact from assumption, all framing elements referring to the way things are or the effects certain actions or situations have were organised into one main category.

Within this last ingredient, we have divided the sub-categories according to the groups or systems the knowledge & convictions are about. Lastly, the lack of knowledge (perceived ignorance) and related knowledge-related uncertainty that people refer to was also designated to this category.

Questions that can be used to elicit knowledge & convictions in conversations are:

- *How are things really? What do you think? What do you know?*
- *What effect do things have? How much effect does what you do have?*
- *Can change really happen? What is hindering change?*
- *Who can you trust and who not?*
- *What do other people want, feel or do? What do they find important? What do they think or know? And what do they think should be done?*

6.1 Knowledge & convictions regarding oneself

The first sub-category of knowledge & conviction topics regards ideas about the effects certain situations will have on the self and (self-)efficacy.

Sub-categories and codes

- Ideas about the impact of eating meat, meat types & alternatives on oneself
    - Impact on personal financial space
    - Ease of purchase of meat, meat types and alternatives
    - Ease of preparation of meat, meat types and alternatives
    - Nutritional necessity (protein, B12, iron, fat, energy)
    - Other intake effects (stress, hormones, antibiotics resistance, GMOs, metals, disease)
    - Impact on (religious) virtue, karma, afterlife
    - Impact on identity, status
- Ideas about the impact of animal farming and slaughter practices (common, alternative) on oneself
    - Link between animal treatment and meat taste
    - Economic impact (livelihood or via national economy)
    - Environmental and landscape impact
    - Impact of farming and slaughter practices on the self
- Ideas about self-efficacy and personal influence on animal farming and meat consumption
    - The impact of personal action on the status quo of animal farming and meat consumption
    - Impact on the norms & behaviour of other humans
    - Impact on the systemic-institutional context
    - Impact on farming systems
    - Impact on farm animals
    - Impact on the environment (ecological footprint)
    - Limitations of impact of one's actions
    - The possibilities and limitations to change oneself
- Perceived lack of knowledge about issues affecting oneself

6.2 Knowledge & convictions concerning humans and culture

The second and by far largest sub-category of knowledge & conviction topics encompasses ideas about the way things are for humans in general or in certain cultures, including the perceived behaviours, values, norms, feelings, interests, and knowledge & convictions of other (groups of) humans.

Sub-categories and codes

- Ideas about mankind in relation to farming and carnivory/onmivory/herbivory
    - Human nature, instinct
    - Human digestive system's suitability for carnivory/onmivory/herbivory
    - History of farming
    - Cultural heritage

- Ideas about the characteristics certain humans possess earning them moral status
    - Relationship, kinship
    - Group membership
    - Power dependency
    - Fairness
    - Species membership
    - Intrinsic value
- Ideas about behaviours of others
    - Peers/loved ones
    - Groups of people/(sub-)cultures
        - General cultural consumption patterns
        - Religious practices
        - Social habits and rituals
    - Stakeholders in the animal production chain (farmers, butchers, transporters, retailers)
        - Farming practices
        - Practices during slaughter
        - Practices during transport
        - Practices in production and sale of protein products
    - The government/policy makers
    - Controllers (food authorities)
    - Social movements/NGOs
- Ideas about values present in human society
    - Peers/loved ones
    - Groups of people/(sub-)cultures
    - Religious groups
    - Stakeholders in the animal production chain
        - Farmers
        - Butchers
        - Transporters
        - Retailers
    - Controllers (food authorities)
    - The government/policy makers
    - Social movements/NGOs
- Ideas about norms in human society
    - Peers/loved ones
    - Groups of people/(sub-)cultures
        - Cultural habits and taboos
        - Religious decrees
    - Stakeholders in the animal production chain (farmers, butchers, transporters, retailers)
    - Controllers (food authorities)
    - The government/policy makers
    - Social movements/NGOs
- Ideas about feelings of other humans
    - Peers/loved ones
    - Groups of people/(sub-)cultures
    - Religious groups
    - Stakeholders in the animal production chain
        - Farmers
        - Butchers
        - Transporters
        - Retailers
    - Controllers (food authorities)
    - The government/policy makers
    - Social movements/NGOs
- Ideas about interests of other humans

  - Peers/loved ones
    - Feeling good
    - Being healthy
    - Living according to values
  - Humans in general
    - Human health necessity
    - Human food necessity
    - Future generations (sustainability)
    - Living according to values
  - Group members (i.e. based on culture, religion)
    - Protect shared group identity and group
  - Stakeholders in animal production chain
    - Earning livelihood
    - Making profit
    - Working circumstances
    - Image (licence to produce)
  - Social movements
    - Defending values, striving for ideal
- Ideas about knowledge & convictions (and lack thereof) of other humans
  - Peers/loved ones
  - Groups of people/(sub-)cultures
  - Religious groups
  - Stakeholders in the animal production chain
    - Farmers
    - Butchers
    - Transporters
    - Retailers
  - Controllers (food authorities)
  - The government/policy makers
  - Social movements/NGOs
- Judgement of trustworthiness and reliability
  - Peers/loved ones
  - Groups of people/(sub-)cultures
  - Religious groups
  - Stakeholders in the animal production chain
    - Farmers
    - Butchers
    - Transporters
    - Retailers
  - Controllers (food authorities)
  - The government/policy makers
  - Social movements/NGOs
- Ideas about the impact of eating meat, meat types and alternatives on other humans
  - Nutritional value of meat, meat types and alternatives (protein, B12, iron, fat, energy)
  - Other intake effects (stress, hormones, antibiotics resistance, GMOs, metals, disease)
  - Impact on (religious) virtue, karma, afterlife
- Ideas about the impact of animal farming and meat consumption (common, alternative) on other humans
  - Employment, livelihood
  - Economy, making profit
  - Health, hygiene, disease
  - Food supply
  - World food distribution & fair trade
  - Ecology, environmental issues (see further: the earth/the environment)
  - Landscape change

    - Civilization level in relation to treatment of animals (see further: knowledge & convictions concerning animals)
- Ideas about what other humans should do (normative statements)
    - Peers/loved ones
    - Groups of people/(sub-)cultures
    - Religious groups
    - Stakeholders in the animal production chain
        - Farmers
        - Butchers
        - Transporters
        - Retailers
    - Controllers (food authorities)
    - The government/policy makers
    - Social movements/NGOs
- Ideas about efficacy and influence of other humans and culture on animal farming and meat consumption
    - History, change in time
        - The move from hunter-gatherer and horticultural societies to agricultural and industrial societies
        - The possibilities and limitations of other humans and culture to change
    - The impact of actions by humans on the status quo of animal farming and meat consumption
        - Impact on the norms & behaviour of other humans
        - Impact on the systemic-institutional context
        - Impact on farming systems
        - Impact on farm animals
        - Impact on the environment (ecological footprint)
        - Limitations of impact of others' actions
- Perceived lack of knowledge about other humans and culture

6.3 Knowledge & convictions concerning the production chain and relevant institutions

The next big sub-category of knowledge & conviction topics encompasses ideas about the way things are in the production chain and relevant institutions.

Sub-categories and codes

- Ideas about availability & proximity of meat, meat types and alternatives
    - Animal species generally eaten
    - Alternatives
    - Supermarket collection
    - Butchers
- Ideas about animal production systems (common, alternative, hunting & fishing)
    - Species commonly farmed
    - Housing
    - Measures and treatment
    - Feed origin and quality
    - Medicine use
    - Life span
    - Differences between farming methods
    - Stunning
    - Speed & numbers
    - Humane slaughter
    - Religious slaughter
    - Species commonly hunted & fished
    - Hunting & fishing methods
    - Fish farming
    - Game farming
- Ideas about government, policy & legislation

    - Policy (national, European, WTO)
    - Legislation, legal system & law-enforcement (control)
    - Subsidies and taxes
- Ideas about food production technologies, food prices, world food supply & distribution issues
    - Food prices (absolute/relative to income)
    - Margins of retail and other actors in the food production chain
    - Export, import issues
    - Transport issues
    - Distribution of food
    - Efficiency of food production
    - Welfare increase
    - Population growth
    - Technology increase in food production (incl. GMOs & in-vitro meat)
- Judgement of information availability & reliability
    - Availability of information (and lack thereof)
    - Trustworthiness of advertising
    - Trustworthiness of product labelling and control
    - Concealment by certain parties
    - Trustworthiness of messages of NGOs
    - Judgement of ambiguity of information
- Ideas about power relations, change and influence of the systemic-institutional context on animal farming and meat consumption
    - Ideas about power relations within the systemic-institutional context
        - Citizens
        - Consumers
        - Producers
        - Veterinarians
        - Government, politicians
        - Retail
        - The system/the market
    - History, change in time until now
        - The possibilities and limitations of the systemic-institutional context to change in the future
    - The impact of possible change of the system on the status quo of animal farming and meat consumption
        - Impact on the norms & behaviour of other humans
        - Impact on farming systems
        - Impact on farm animals
        - Impact on the environment
        - Limitations of impact of a change in the system
- Perceived lack of knowledge about the production chain and relevant institutions

6.4 Knowledge & convictions concerning animals

The penultimate sub-category of knowledge & conviction topics encompasses ideas about the way things are with regards to animals, including their perceived feelings, and interests.

Sub-categories and codes

- Ideas about the characteristics certain animals possess earning them worthy of consideration of interests (moral status)
    - Species differentiation
    - Kinship
    - Relational/emotional value
    - Intelligence
    - Ability to feel pain/suffer
    - Ability to experience emotion
    - Intrinsic/inherent value

    - Indirect worth to humans (instrumental value)
- Ideas about interests of animals
    - Recognition of intrinsic/inherent value
    - Protection of integrity
    - Physical well-being (health, absence of discomfort and suffering)
    - Emotional well-being
    - Naturalness / being able to perform natural behaviour
    - Able to fulfil capabilities of species
    - Satisfaction of instincts
    - Species continuation
    - Staying alive
    - Humane slaughter
    - Respect from humans
    - Love, care
    - Service/sacrifice of life to humans/God
- Ideas about the feelings of animals
    - Comfort
    - Pleasure
    - Contentment
    - Fear
    - Suffering
    - Boredom
- Ideas about the impact of different production systems (common, alternative) on animals
    - Species commonly farmed
    - Housing
    - Measures and treatment
    - Feed origin and quality
    - Medicine use
    - Breeding and selection
    - Genetic modification
    - Life span
    - Animal behaviour in different production systems
    - Animal welfare impact
- Ideas about the impact of manners of slaughter on animals
    - Stunning
    - Speed & numbers
    - Humane slaughter
    - Religious slaughter
    - Animal behaviour in different slaughter systems
    - Animal welfare impact
- Ideas about the impact of hunting & fishing on animals
    - Species commonly hunted & fished
    - Hunting & fishing methods
    - Fish farming
    - Game farming
    - Animal welfare impact compared with animal production systems and slaughter
- Ideas about animals and animal species in relation to farming and carnivory/omnivory/herbivory
    - Reference to animals also killing and eating meat
    - Merits and downsides of animal farming and meat consumption for certain animal species
    - Role of animals for humans
    - Role of animals in/for the world
- Perceived lack of knowledge about animals

6.5 Knowledge & convictions concerning the earth/environment

The last sub-category of knowledge & conviction topics people refer to when constructing their everyday-life framing of animal farming and meat consumption, deals with the way things are with regards to the earth/biosphere and environmental issues.

Sub-categories and codes

- Ideas about the characteristics the earth/environment possesses earning it moral status
  - Relational/emotional value
  - Intrinsic value
  - Indirect worth to humans (instrumental/sustenance value)
- Ideas about the fragility/resilience of nature and life in general
- Ideas about the environmental impact of different animal farming and meat consumption (common, alternative) on the earth/environment
  - Land use
  - Rain forest degradation
  - Climate change
  - CO_2/methane/fine particles exhaust
  - Fertilizers
  - Water use
  - Ecology, biodiversity
  - Overfishing
  - Ocean floor scraping
  - Genetic modification
- Perceived lack of knowledge about the earth/environment
